# Brazilian livestock extensionists attitudes toward different fates for male dairy calves

**DOI:** 10.3389/fvets.2025.1448756

**Published:** 2025-01-31

**Authors:** Karolini Tenffen De-Sousa, Juliana Bernardes Benetton, Luiz Carlos Pinheiro Machado Filho, Maria José Hötzel

**Affiliations:** Laboratório de Etologia Aplicada e Bem-Estar Animal, Departamento de Zootecnia e Desenvolvimento Rural, Universidade Federal de Santa Catarina, Florianópolis, Brazil

**Keywords:** advisors, animal welfare, ethics, extension, surplus calves

## Abstract

Male calves are not economically attractive for most dairy farms in the current system and are therefore considered surplus in the dairy industry. The issue has gained growing attention in many countries, due to economic and animal welfare implications. It is known that livestock extensionists play a crucial role in facilitating change and promoting sustainable practices among the farming community. This study aimed to investigate the knowledge and attitudes of Brazilian dairy extensionist regarding three potential solutions to the issue of male dairy calves: the use of sexed semen, rearing for beef, and culling at birth. Livestock extensionists from different regions of Brazil (*n* = 344) participated in an online survey and answered closed (multiple choice and Likert scale) and open questions. Most participants (63%) were male, 61% with ages ranging from 19 to 35 years old and 49% held a graduate education in veterinary medicine. The practice of culling male calves was generally rejected as unethical, while the use of sexed semen to reduce the birth of males was seen as having positive ethical (75%), environmental (77%), and animal welfare (81%) implications. However, participants considered it difficult to implement in small farms due to its high cost. Raising male calves for beef production was perceived as more economically viable for small and medium size (49%) than for large dairy farms (32%). In general, participants considered the fate of male dairy calves a complex issue, suggesting the need for more than one alternative to address it effectively. However, extensionists acknowledged not discussing the topic with farmers, indicating a potential lack of awareness or interest in the issue. It is possible that participants may not have received sufficient training or engaged in discussions on animal welfare issues, as this is a relatively new subject during their professional development. The nascent nature of the issue in Brazil emphasizes the need for comprehensive education and knowledge exchange initiatives in the industry, to ensure that practitioners are well-prepared to adopt best practices in male calf management. This may foster local discourse on the topic and the development of contextualized knowledge, a crucial step for addressing aspects of dairy farming sustainability.

## 1 Introduction

The dairy production chain has undergone significant transformations over the years, driven by efforts to enhance efficiency and productivity. During decades of intensification processes, dairy production transitioned from small and family farming systems to large-scale operations, accelerating the adoption of advanced technologies (1, 2). Notably, the genetic selection of highly productive and specialized breeds has become widespread (3). Alongside these advancements, changes in the milk production model have introduced some ethical challenges concerning the treatment of dairy cattle. Many aspects of the production systems are at odds with public values, with animal welfare emerging as a primary public concern around the world (4–5).

Dairy farms typically aim to impregnate cows and have a calf every year to maintain high milk production levels. This results in millions of female and male calves being born worldwide yearly. While female calves have the potential to be used for replacements and to expand the dairy herd, males contribute minimally to dairy farm profitability. Consequently, they are considered surplus to the dairy industry, as their beef production traits have diminished due to selection for higher milk yield (7). In New Zealand, approximately 40% of male dairy calves are considered a by-product and are transported to slaughter when they are between 4 and 7 days old (5). Similarly, around 22% of male dairy calves in the United Kingdom are slaughtered shortly after birth (9). Research conducted in Brazil has shown that approximately 23% (10) or 35% (11) of farmers killed male dairy calves soon after birth. Various alternatives to addressing the issue of surplus male dairy calves have been proposed. These include culling calves immediately after birth, raising them for meat production, or employing sexed semen technology to decrease the number of male calves born on dairy farms (12, 13).

The fate of male calves in the Brazilian dairy industry is often overlooked, with limited discussion among industry professionals. This lack of discussion is compounded by outdated information and farmers’ low receptivity, which can hinder the effectiveness of professional interventions. Reports from dairy farmers in Canada (14) indicate that veterinarians have little interest and involvement in calf health and rearing. Thus, it is important to explore the knowledge and viewpoints of professionals engaged in the dairy chain to expand the debate on the fate of male calves in the dairy system. To advance sustainability in the dairy sector, understanding the perspectives and motivations of professionals working directly with farmers is essential. This insight can inform the development of collaborative practices among stakeholders, leading to more effective policies and management strategies to improve animal welfare and foster a sustainable dairy industry (15, 16). This study aimed to investigate the knowledge and attitudes of Brazilian livestock extensionists regarding three potential fates for male dairy calves: the use of sexed semen, raising for beef, and culling at birth.

## 2 Methodology

Research involving humans in Brazil is guided by Regulations n. 466/2012 and 510/2016 of the Brazilian National Health Council. The Regulation 510/2016 specifies that surveys involving public opinion that do not identify participants are not subject to review by the CEP/CONEP (*Comissão Nacional de Ética em Pesquisa*). All procedures followed the ethical principles outlined in these regulations. Prior to completing the questionnaire, participants were required to read an informed consent statement and accept the conditions of the study. These conditions included anonymity, voluntary participation, and the use of data solely for scientific research purposes. Participants were assured that their responses would be treated confidentially and that they could withdraw from the study at any time by not submitting the form. All participants were required to give consent about their participation by clicking a button saying, “I agree to participate in the research” before taking the survey”.

### 2.1 Participants’ recruitment

Brazilian professionals involved in dairy extension — individuals with agricultural education who provide various services such as production advice and technology transfer — were invited to participate in an online survey conducted anonymously and voluntarily between January and April 2021. These professionals included those from public sectors (through government projects or funding institutions) and private companies (where farmers pay directly for advisory services).

The survey was administered using Google Forms and distributed exclusively online through multiple channels, including social media platforms (Facebook and Instagram), email, and WhatsApp. The survey link was shared in groups and on personal pages, specifically targeting individuals working with dairy extension. Additionally, an exponential nondriscrimination snowball sampling (17) was employed, wherein each participant was encouraged to share the survey with their network (18). Before answering the questionnaire, participants were provided with a brief explanation regarding the anonymity of their responses and the option to withdraw from the survey at any time by closing the form. Subsequently, each participant was required to indicate if they agreed with the consent form, and only participants who agreed were directed to complete the survey.

### 2.2 Questionnaire structure

The questionnaire was divided into five sections including multiple-choice, 5-point Likert scale, and open questions. Section 1 contained sociodemographic questions (sex, age, region of current work, undergraduate or graduate education, years of experience, current activity, and work location). Participants were asked whether they interacted with dairy farmers in their activity, with response options including “yes,” “no,” “only sporadically,” and “only indirectly.” Only participants indicating interaction with dairy farmers proceeded to the next section. In section 2, participants were asked about their perceptions regarding the predominance of practices concerning the fate of male dairy calves on Brazilian dairy farms; they were asked to identify the most common method employed for culling male calves and to assess how the care provided to male calves compared to that given to female calves.

In section 3, each participant was randomly assigned to one of three potential fates for male dairy calves: the use of sexed semen, raising for beef, and culling at birth. Following, nine questions were (all on five-point Likert scale) designed to assess participants attitude (19): How much do you disagree/agree (1 = strongly disagree to 5 = strongly agree) with the following statements: “It is an economically viable alternative for small (up to 30 milking cows) and medium (range from 31 to 100 milking cows) dairy farms”; “It is an economically viable alternative for large-sized (more than 100 milking cows) dairy farmers”; “It is an acceptable alternative from an ethical point of view”; “It is a viable alternative from a technical point of view”; “It is an adequate alternative from an environmental point of view”; “It is an adequate alternative from an animal welfare point of view”; “It is discussed among milk chain professionals”; “There is sufficient technical knowledge available to assist dairy farmers with this alternative”; and “Dairy farmers are interested in this alternative.” Participants were then invited to answer two open-ended questions, one about the main problem or limitation they perceived regarding the practice and one asking their suggestions to improve the issue of male calves in dairy farms.

In section 4, participants were asked to rate, using a 5-point Likert scale (1 = low impact to 5 = high impact), the potential impact of various factors on the sustainability of the dairy supply chain in the coming years. The factors presented were milk produced artificially in the laboratory, alternative products or plant-based substitutes to cow’s milk, the increase in vegetarian and vegan population, changes in environmental legislation and stricter inspections, legislative changes aiming at improving animal welfare, concerns about zoonoses, increased consumer demand regarding environmental issues and increased demand regarding animal welfare. Following this, participants were asked for their opinions on increased consumer demands regarding animal welfare issues in an open-ended question.

In the last section of the questionnaire, an additional question (also using a Likert five-point scale) assessed potential reasons for attitudes based on an animal welfare construct of affective states: “In your opinion, calves are able (1 = totally able to 5 = totally unable) to feel pain, fear, happiness, boredom?”; “In your opinion, should calves be free from hunger and thirst; pain, injury, or disease; discomfort; fear and stress; and free to express natural behaviors?.” Additionally, participants were requested to list in an open-ended question the most important factors to promote the welfare of a dairy herd.

### 2.3 Data analysis

We received a total of 434 responses, 90 of which were eliminated because the participants reported not interacting with dairy farmers in their work or identified themselves as dairy farmers. This resulted in a final sample of 344 complete questionnaires, distributed across the three potential fates for male dairy calves: the use of sexed semen (*n* = 119), raising for beef (*n* = 126), and culling at birth (*n* = 99). Our study was based on a convenience sample and should not be considered representative of all Brazilian professionals. However, they provide a rich overview of the knowledge and perspectives of livestock extensionist from various regions and with different training backgrounds, working in both public and private settings. The variation in the number of participants for each potential fate is a result of the exclusion of some participants. Thus, we used a qualitative approach to generate data to interpret and understand the knowledge of participants (20, 21) and a quantitative approach to identify differences in participants’ attitude toward the general theme “Different fates for male dairy calves: the use of sexed semen, raising for beef, and culling at birth”.

The qualitative approach was based on reflexive thematic analysis and the semantic codes was generated following the deductive orientation (22, 23). The analysis involved carefully reading all responses multiple times to fully understand the content, followed by coding based on the themes of the questionnaire (practices, limitations, animal welfare, and consumers). This coding process helped organize the data, reduce complex responses into manageable categories, and highlight key patterns and insights.

After that, the interpretation phase involved rereading the responses to make sure that the ascribed meanings made sense and editing as needed to improve reliability. For the quantitative approach, a descriptive analysis was used to represent the percentage of responses to multiple-choice and 5-point Likert scale questions. For results presentation, questions with the five-point Likert scale were reclassified into three points (e.g., 1 and 2 = negative, 3 = neutral, and 4 and 5 = positive). The number of responses for the open questions varied because some responses that were not considered valid were excluded from the analysis.

## 3 Results

Table 1 summarizes participants’ sociodemographic characteristics. The survey covered all Brazilian regions, with a predominance of participants from the South and Southwest regions. Most participants were male, aged between 26 and 35, and held a graduate education. The majority were professionals involved in field activities.

**Table 1:** Profile of the 344 Brazilian dairy chain professionals who participated in the survey on knowledge and attitude to different fates of male dairy calves.

Variables	*N* of participants	%
Gender		
Female	126	37
Male	218	63
Age (years)		
18 to 25	64	19
26 to 35	145	42
36 to 45	75	22
46 to 55	31	9
56 to 65	25	7
66 and over	4	1
Region of current work		
South	152	44
Southwest	117	34
North	8	23
Northwest	44	13
Center-West	23	7
Undergraduate training		
Technical level	14	4
Veterinary medicine	168	49
Agronomy	38	11
Animal science	105	30
Other	19	5
Years of experience		
0 to 5	115	33
6 to 10	94	27
11 to 15	45	13
16 or more	90	26
Current activity		
Field professional	177	51
Researcher	29	8
Academic	25	7
Student	48	14
Other	65	19
Location of work		
Public company	72	21
Private company	113	33
University	70	20
Self-employed	50	15
Dairy industry or cooperative	39	11

### 3.1 Participants’ perception of currently male calf management on dairy farms

**Figure 1:**
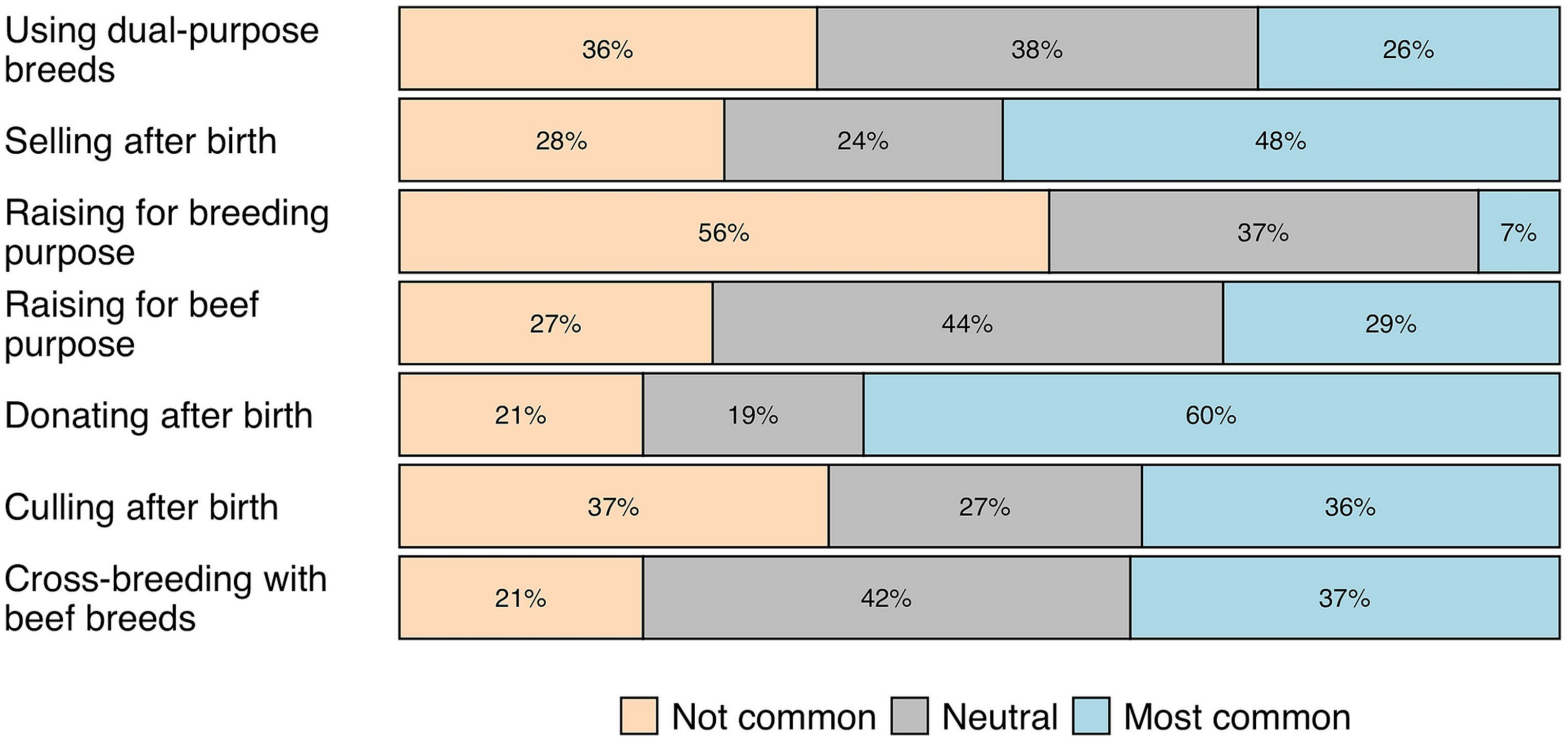
Participants’ (*n* = 344) perception (not common, neutral, and most common) of the predominance of seven possible fates to address the issue of male calves on Brazilian dairy farms.

When questioned about the predominance of seven possible fates for male dairy calves, 60% of all participants (*n* = 344) indicated that donation to third parties (e.g., to neighbors or acquaintances) occurred in most dairy farms (Figure 1), while 56% considered raising male dairy calves for breeding purposes not common.

Forty-five percent of participants were unaware of the practices used in dairy farms for culling male calves. According to 38% of participants, culling is performed by blunt force trauma without anesthesia, while only 4% indicated that it is done with anesthesia; 10% mentioned sale or donation, and 3% mentioned other practices such as colostrum deprivation, leaving the calf to die, stabbing in the heart, and injections (CB30, lidocaine, and xylazine).

Participants perceived differences in management practices between male and female dairy calves. The primary distinction mentioned was in milk feeding, with 65% of participants stating that male calves received less milk than females. Furthermore, 41% stated that colostrum management, 38% housing, and 14% navel care is inferior to male calves.

### 3.2 Participants’ opinions regarding the use of sexed semen, raising calves for beef purpose and culling after birth

The majority of participants (66%; *n* = 344) said on the open-ended question that raising male calves for beef purpose is one of the best solutions to manage male dairy calves. Additionally, 42% of participants mentioned sexed semen as the best solution. However, 37% of participants emphasized that at least two alternatives need to be combined to manage male calves in dairy farms, while 13% stated that the decision on which practices to adopt depends on each farms’ individual conditions. Few participants mentioned culling after birth (7%) or using male calves for breeding purposes (6%) as a solution for male calves issue.

**Figure 2:**
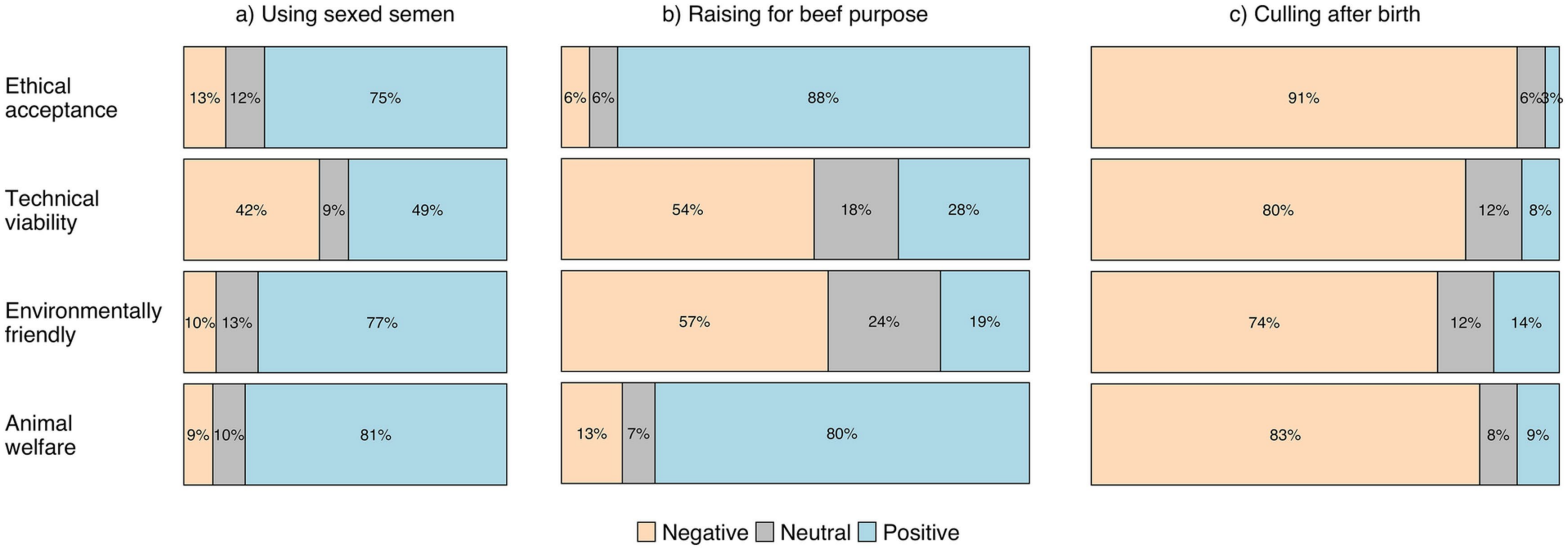
Participants’ attitudes (negative, neutral and positive) to three potential fates for male dairy calves: **(A)** using sexed semen (*n* = 119), **(B)** raising for beef purpose (*n* = 126) and, **(C)** culling after birth (*n* = 99) presented in the survey from the ethical acceptance, technical viability, environmentally friendly, and animal welfare point of view.

Regarding using sexed semen, participants expressed a positive attitude toward all evaluated aspects, though more had negative attitude to technical viability (Figure 2A). Fifty four percent of the participants considered the use of sexed semen as an economically viable alternative for farms of various size (small, medium, and large). However, opinions concerning the farmers’ interest varied, with 32% believing that farmers are interested in this practice and 32% believing that they are not. According to 45% of the participants, professionals do not discuss the use of sexed semen, and for 44% there is no sufficient technical knowledge available to assist farmers on this alternative. In the open-ended response, 66% of participants mentioned the low efficiency and high cost as the main limitation of the adoption of sexed semen.

Participants who responded to questions about raising male dairy calves for beef purposes were more positive about ethical acceptance and animal welfare than about technical viability and environmentally friendliness (Figure 2B). The practice of raising male calves was perceived as more economically viable for small and medium-sized farms (49%) compared to large dairy farms (32%). Fifty-three percent of participants stated that dairy farmers are interested in raising male calves, but 52% mentioned that this alternative is not discussed among milk chain professionals. Additionally, 47% agreed that there is no sufficient technical knowledge available to assist farmers with this alternative. In the open-ended question, most (55%) participants did not have a clear position regarding raising male calves for beef and cited economic feasibility (48%) and lack of space (27%) as the main limitations of this practice.

Almost all of participants rejected the practice of culling male calves after birth (Figure 2C). When this practice is adopted, participants believe that culling male is more economically viable for large farms (77%) than for small and medium farms (37%). Sixty-six per cent of participants agreed that dairy farmers are not interested in culling male calves, and neither are professionals, since most (73%) believe that professionals do not discuss this practice. Similar to the other practices, 45% of participants stated that there is no sufficient technical knowledge available to assist farmers with this alternative. Participants also mentioned in open-ended question concerns that culling the male calves is unacceptable (63%), and that ethical (14%) and animal welfare (19%) are issues limiting this practice.

### 3.3 Attitudes to animal welfare

**Figure 3:**
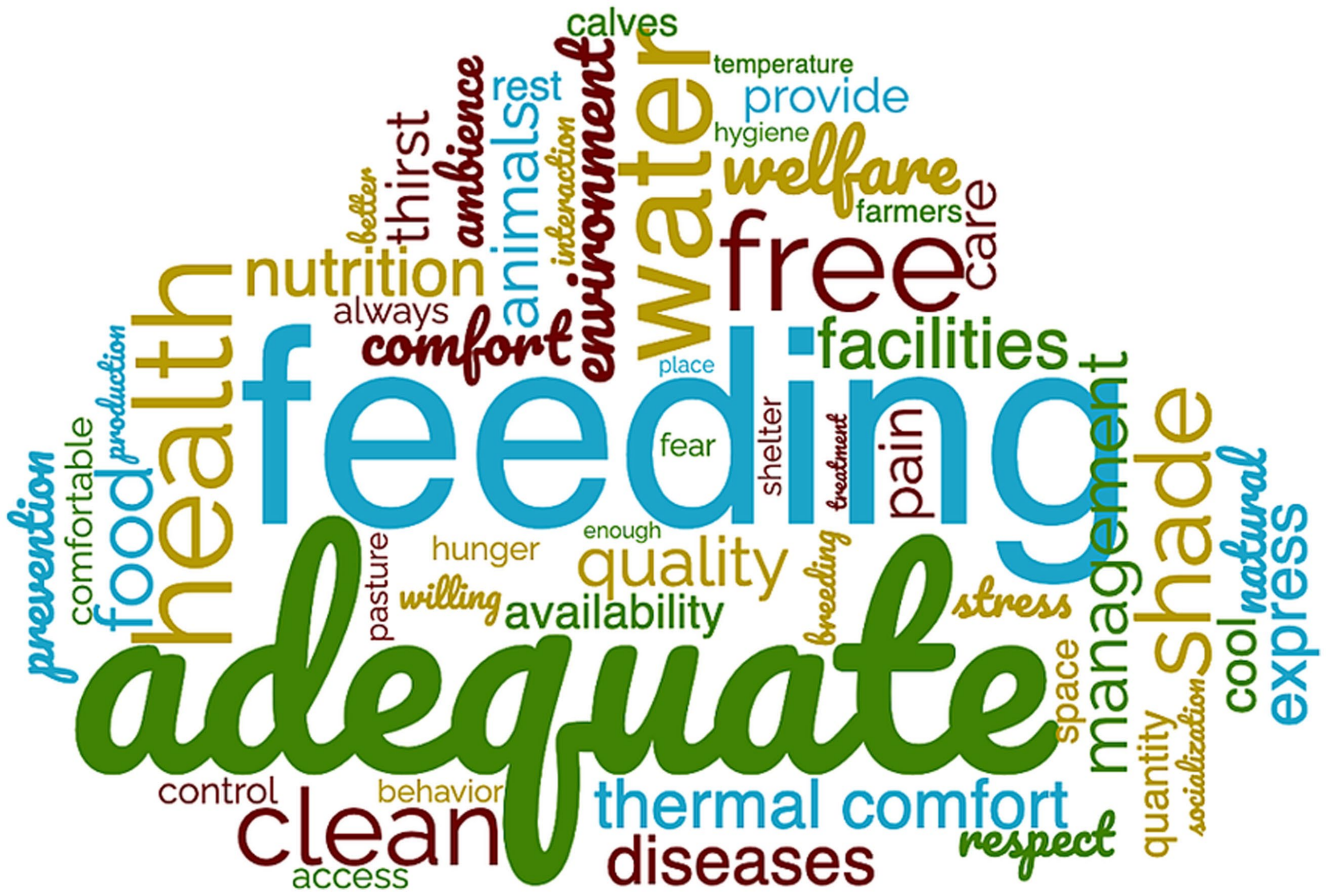
Word cloud generated using the 60 most frequently used words in response (*n* = 344) to the question “In your opinion, what are the most important elements to promote animal welfare in a dairy herd?” The words appearing in larger type were used more frequently (threshold ≥10).

Most of the participants (*n* = 344) believed that calves have the ability to feel emotions (pain: 96%, fear: 93%, joy: 90% and boredom: 84%). This led to a consensus that calves should be free from negative experiences (hunger and thirst: 96%; pain and disease: 99%; discomfort: 94%; fear and stress: 93%) and free to express natural behaviors (92%). We used a word cloud (Figure 3) to capture the main elements cited by participants on the open-ended question about dairy cattle welfare.

### 3.4 Sustainability of the dairy sector in the coming years

**Figure 4:**
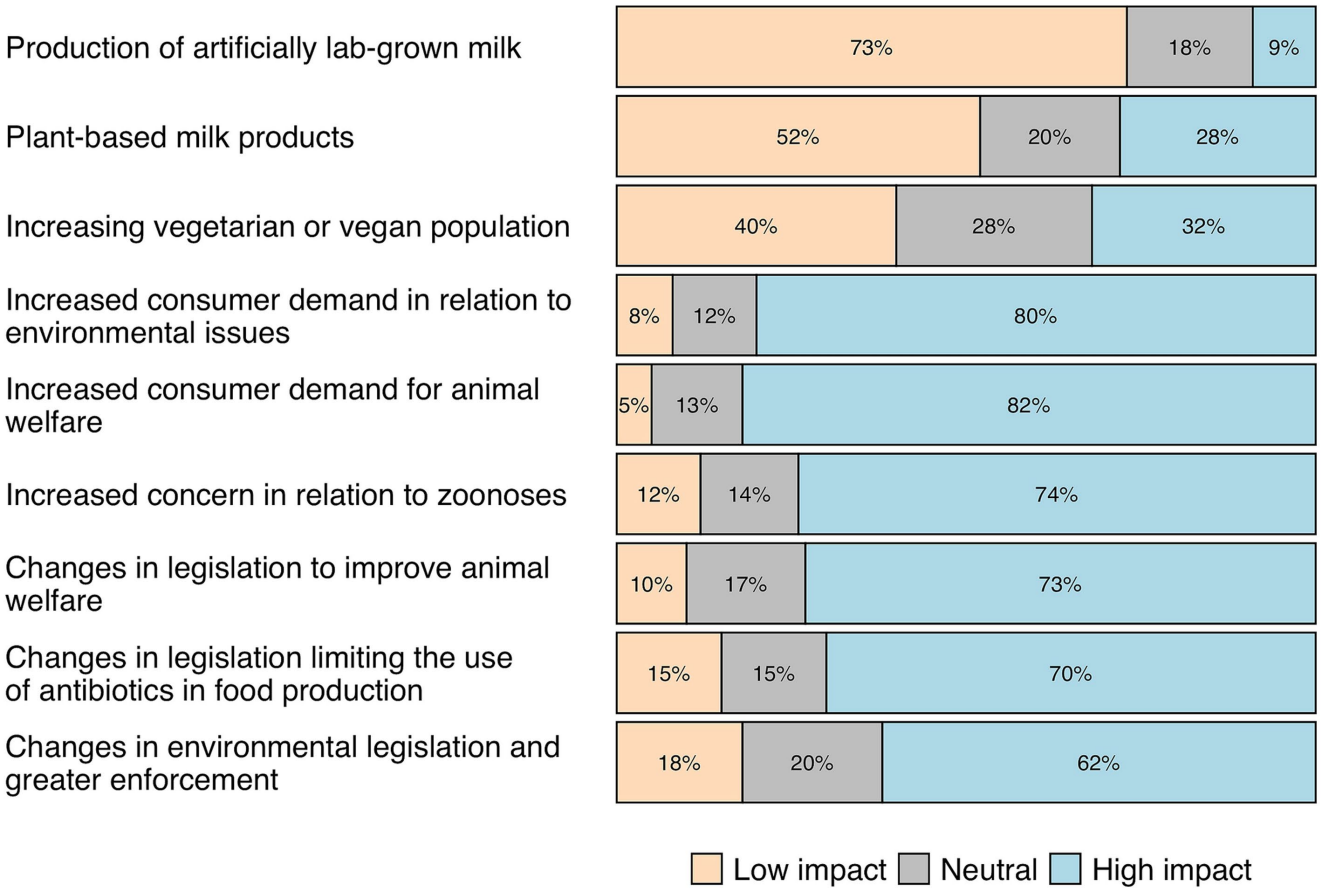
Participants’ (*n* = 344) opinions regarding factors that may impact the sustainability of the dairy supply chain in the coming years.

Participants (*n* = 344) indicated that the rise in public interest in animal welfare and environmental issues would have the most significant impact on the sustainability of the dairy supply chain, while alternative products would have the least impact (Figure 4). In the open-ended question, 39% of participants mentioned an increasing concern among consumers regarding the livestock system. However, 18% believe that consumers have limited knowledge about livestock system, and 6% suggested the need to improve communication with consumers. Thirteen per cent of participants believed that consumer concerns could drive changes in the livestock system, and 12% believed that consumers could influence their purchasing decisions.

## 4 Discussion

The Brazilian livestock extensionists participating in this survey were aware of the practices used on farms in Brazil to deal with male dairy calves. They also recognized the urgency to change practices to address ethical, economic and environmental issues involved in the surplus male calf problem. Most agreed that a combination of management practices is necessary to effectively address the current scenario, which includes a significant proportion of calves being culled at birth. This aligns with academic discussion of the issue of the surplus dairy and its recognition as a wicked problem (13). However, our findings suggest that these professionals must be better prepared to effectively assist dairy farmers in changing the male calf scenario in dairy farms. This requires an increased understanding of the current discussion and technical developments in the area and a readiness to engage in meaningful conversations with farmers.

Participants reported that male dairy calves are viewed as a low-value or waste products in the dairy industry, which affects decisions regarding those animals. Some examples they described are the inferior quality of care given to male calves compared to that given to female calves and the decision to cull male calves immediately after birth. These practices have come under public criticism and pose a threat to the sustainability of the dairy industry (4, 13). Confirming participants’ perceptions, the practice of culling male dairy calves is common on Brazilian dairy farms, and it is carried out in a manner that contradicts principles of humane care (10, 11). Despite holding negative attitudes toward culling male calves, participants acknowledged not discussing the practice, which is concerning given its widespread use on dairy farms. Aside from welfare implications for the animals due to the lack of humane methods, killing surplus male calves raises moral concerns when the calf is perceived as a product or surplus waste, undermining its intrinsic value (24, 25). Some reasons farmers allege for culling newborn male dairy calves are a shortage of on-farm infrastructure needed to raise the animals in farms that are specialized for dairy and the high costs of feeding and labor involved in their management (11, 14). Dairy farmers interviewed in southern Brazil reported that they culled newborn male calves out of necessity rather than choice (11), and worried about other people’s reactions if they became aware of this practice (26). This highlights the importance of fostering dialog between farmers and extensionists to help finding the best solutions for the surplus male problem.

A concerning finding related to the issue of culling surplus male calves was that nearly half of the participants were unfamiliar with the culling methods used on the farms. Those who were aware reported that it is typically done by blunt force trauma without anesthesia, consistent with findings reported in on-farm studies in Brazil conducted by Hötzel et al. (11) and Fruscalso et al. (10). The use of this method has been also reported in other countries (14, 27, 28) and is considered inhumane by the Brazilian Council of Veterinarians (29) and the American Veterinary Medical Association (30). Possibly due to its controversial nature, discussing the details of calf culling is a seldom-addressed and delicate subject, as reported by participants in this study and farmers interviewed by Rodrigues et al. (26). The reluctance to openly discuss this issue, possibly in anticipation of criticism, overlooks the fact that culling surplus males, regardless of the method, contradicts public values (31, 32).

A common practice reported by the participants was the donation of male calves soon after birth to farmers in the region. Donation to neighbors or acquaintances was cited by farmers as a solution to surplus males (11). In regions of Brazil that are experiencing growth and intensification of the dairy industry, the tendency is for the surplus of male calves to increase (33), limiting the effectiveness of this practice as a solution to the problem. However, donations to rural producers who do not raise cattle can be an interesting alternative in regions with small dairy farms, where establishing a production chain around surplus calves may be more challenging. If these calves are raised for subsistence, this may contribute to local food security. However, challenges may arise in both scenarios due to the informality inherent in this activity, leading to issues such as the lack of adequate conditions for the transportation of these calves and legal impediments associated with moving animals between farms. Additionally, donations may be associated with inadequate housing, feeding, and animal health care. For example, on the birth farms, ensuring a healthy and well-nourished animal may not be a priority and calves may not receive an adequate supply of colostrum and feed. Participants described lower levels of care for male than female newborn calves, who are usually retained on the farm for herd replacement a phenomenon also reported in other countries [e.g., (26)]. On the destination farms, the quality of care may depend on the technical and financial conditions to keep the animal. The association of donation with a low-value product may exacerbate issues in both scenarios. In another study, Brazilian dairy farmers reported donating calves to avoid culling and expressed concerns that calves may not receive proper treatment in the destination farms (34), highlighting the need to address the problems associated with culling and donation together.

Sexed semen emerged as the most suitable solution to address the challenge of male calves born on dairy farms. Most participants expressed positive attitudes to its use when evaluating the ethical, animal welfare, and environmental impacts. Participants mentioned the use of sexed semen to decrease the birth of male calves coupled with the use of dual-purpose breeds and beef semen to direct dairy male calves to beef production. However, opinions regarding the interests of farmers in the technique and its economic and technical feasibility varied, with many noting the high cost and low efficiency in conception rates as primary limitations. Besides reducing the number of unwanted male calves, one of the main advantages of sexed semen is guaranteeing a significant number of replacement heifers for the herd (35). However, the potential disadvantages include an excess of heifers, which may surpass the need for herd replacement, thus recreating the issue of surplus calves. An oversupply of replacement heifers can significantly increase raising costs for dairy farms, leading to financial loss (36). The oversupply of female calves has become a prominent topic of discussion (13, 36) underscoring the importance of exploring alternative solutions to minimize reputational risks for the dairy industry (16). In Brazil, this may not be an immediate concern, given the ongoing expansion of the national herd. Nonetheless, the limited mention of this issue by participants in this study suggests a low level of familiarity with the technical discussion on the topic.

Participants’ attitudes to raising male dairy calves at the dairy farm were conflicting. Although, in general, they agreed that raising male calves at the dairy farm is an acceptable alternative from an ethical and welfare point of view, more than half of the participants did not see it favorably from a technical and environmental perspective. Similar to the use of sexed semen, there was no consensus among participants regarding raising the male calves. Rearing male dairy calves for beef is considered an efficient way of producing meat and one of the future profitable models for beef production (37). For example, nearly half of the beef produced in the United Kingdom originates from the dairy herd (37) and, in France, some dairy calves are raised as young bulls while others are dairy/beef crossbreds (38). Additionally, in the United States there has been an increase in the use of beef semen in dairy herds from 18 to 26% between 2019 and 2021 (39). Sourcing meat from dairy cattle may improve environmental efficiency (40). It has been shown that beef sourced from dairy herds may have a lower overall carbon footprint than beef from conventional beef herds (41, 42). This is due to dairy beef production’s ability to yield multiple products, including meat, milk, and calves, distributing emissions across these different outputs (42). Hence, rearing male dairy calves for beef production could be an attractive option, particularly when considering the environmental benefits for the long-term sustainability of the dairy industry. However, changes in animal agriculture to reduce global greenhouse gas emissions must avoid unintended consequences with implications for One Health and animal welfare (43). It is well recognized that the success of rearing bull calves for meat is contingent on high nutrition and health standards from birth until their final destination, including antimicrobial usage considerations. These issues, which are highly studied and debated, include inadequate care at various stages, such as on the birth farm, at auction places, during rearing, transportation, and at the destination site where calves will be raised (12, 40, 44–46). Surplus calves raised for meat often experience poor welfare, characterized by high mortality and morbidity rates at calf-raising facilities (45, 47). However, participants did not raise any of these issues in their response to the open questions, suggesting a potential lack of awareness or interest in the care and handling of calves raised for meat. Without a comprehensive understanding of the potentials and limitations of this practice, extensionists will not be equipped to facilitate a transition from culling/donation to raising calves for meat.

The relatively low awareness among extensionists of technical aspects concerning the management of male dairy calves may hinder their role as agents of change. This issue is not limited to male dairy calves; Brazilian livestock extensionists also exhibit a lack of knowledge about optimal tree arrangements for different farm conditions, which hinders the adoption of silvopastoral systems (48). However, studies have shown that dairy farmers value the advice provided by their herd veterinarians or extension professionals (49, 50). Extensionists (51) and farmers (52) in southern Brazil highlighted insufficient discussion of controversial dairy practices as a reason for low adoption of best practices in the case of disbudding dairy calves. Concerns about losing trust among farmers and complying with social norms are potential reasons for extensionists to refrain from discussing with farmers alternatives to on-farm culling of the surplus calves. Extensionists interviewed in the south of Brazil about the practice of dehorning dairy calves believed that colleagues in the profession and the farmers they served approved of their choice of dehorning method and expressed concern that diverging from the farmers’ farming goals (which they believed to be production-oriented) could potentially jeopardize their trust (51). The perception of a lack of technical knowledge to address the issue of surplus male calves may contribute to the absence of discussion regarding the topic, acknowledged by participants. Despite the global importance of the issue and public concern (31, 32), discussions about the fate of surplus calves on Brazilian dairy farms is still in the early stages. Publications reporting study results and recommendations on practices to deal with surplus dairy males primarily originate from countries with established production chains, where discourse on animal welfare and surplus calf management is facilitated [see reviews (12, 47)]. The predominance of scientific articles and international news on the subject published in English may pose a linguistic barrier to greater familiarity with advanced approaches used in other countries, thereby hindering their adoption domestically. Another reason for the low awareness and discussion on the subject may be that professionals within the Brazilian dairy industry who participated in this survey may not have received sufficient training or engaged in discussions on this relatively new topic during their professional development. The nascent nature of the issue in Brazil underscores the need for comprehensive education and knowledge exchange initiatives in the industry. These initiatives can ensure that professionals, who play a central and valued role in the dairy chain, are better prepared to address emerging challenges and help farmers adopt best practices for dealing with surplus male calves. This may facilitate local discussions on the topic and the development of contextualized knowledge, which is a crucial step in addressing dairy farming sustainability. For example, adopting confined systems used and developed in other countries to address the problem may have unintended consequences with animal welfare and One Health (45). Professionals in the dairy chain play a central role in identifying problems, finding alternatives, and supporting dairy farmers. Our findings reveal a need for more in-depth knowledge and open debates among professionals on issues related to the fate of male dairy calves. This is essential to facilitate the development of public policies, guidelines, and protocols to improve the care and handling of male dairy calves from birth, during transport, and until their final destination.

We also sought the participants’ insights into the future of the dairy chain, considering various challenges it faces. The primary challenges identified for the sustainability of the Brazilian dairy industry were animal welfare concerns and the need to comply with related legislation. Studies have shown that public concern regarding the welfare of farmed animals (53), which aligns with the participants’ perception of consumer concerns about animal welfare. Social media facilitates the dissemination of knowledge and information, resulting in consumers’ interest in understating the origin of the food they consume, particularly with regard to animal welfare (54). One example of this is the growing attention given to the fate of dairy calves in both social media and newspapers in recent years (55). As a result, public opposition to dairy practices could undermine its “social license to operate,” with significant financial implications for the dairy industry (56). Public dissatisfaction with current management practices in the dairy industry in Brazil (4) indicates the need for improvements. Participants recognized consumers as catalysts for change in animal production, which is aligned with the assertion that considering public concerns is crucial for finding sustainable solutions to the challenges faced by the dairy industry (13). Another emerging issue of significance is the rise of alternative products or substitutes for cow milk driven by growing concerns about the environmental and human health impacts of animal-sourced proteins, including risks of zoonoses and antimicrobial resistance (43). While participants acknowledged these threats, few believed the supply of alternative products or milk substitutes and the increasing vegetarian and vegan population would significantly impact the sustainability of the dairy supply chain in the coming years. This perception may be influenced by the position of the dairy industry in Brazil, which is still expanding to meet domestic demand, unlike the thriving export beef, poultry, and pork industries. This expansion may create a perception that there will be continued demand for milk, despite the estimated 14% proportion of vegans or vegetarians and Brazil’s large and growing alternative protein market (53).

## 5 Conclusion

Brazilian livestock extensionists are aware of the challenges associated with surplus male calves but require better preparation to effectively support dairy farmers in managing this issue. While the practice of culling male calves was widely considered unethical, extensionists showed a positive attitude toward the use of sexed semen as a strategy to reduce the birth of male calves. However, their attitudes on raising male dairy calves on dairy farms were conflicting. Although they acknowledged that raising male calves on-farm is acceptable from an ethical and welfare perspectives, they considered it less favorably from technical and environmental perspectives.

Overall, the fate of male dairy calves is a complex issue that demands multiple solutions for effective management. The lack of open dialog on controversial topics, such as the culling of male calves, highlights the importance of fostering discussions about animal welfare within the dairy industry. To address this, it is essential to provide continuous education, specialized training programs, and updates on emerging issues to professionals in the field. These efforts would enhance extensionists’ knowledge and attitudes toward current practices, ultimately improving the welfare of male dairy calves and addressing other contentious issues within the industry.

## Data Availability

The raw data supporting the conclusions of this article will be made available by the authors, without undue reservation.
